# Biological evaluation and *in silico* studies of novel compounds as potent TAAR1 agonists that could be used in schizophrenia treatment

**DOI:** 10.3389/fphar.2023.1161964

**Published:** 2023-04-21

**Authors:** Yunjie Wang, Zhaofeng Liu, Jing Lu, Wenyan Wang, Lin Wang, Yifei Yang, Hongbo Wang, Liang Ye, Jianzhao Zhang, Jingwei Tian

**Affiliations:** ^1^ Key Laboratory of Molecular Pharmacology and Drug Evaluation (Yantai University), School of Pharmacy, Ministry of Education, Collaborative Innovation Center of Advanced Drug Delivery System and Biotech Drugs in Universities of Shandong, Yantai University, Yantai, China; ^2^ School of Public Health and Management, Binzhou Medical University, Yantai, China; ^3^ College of Life Sciences, Yantai University, Yantai, China

**Keywords:** trace amine-associated receptor 1, anti-schizophrenia, security, molecular modeling, biological evaluation

## Abstract

**Introduction:** Schizophrenia is a serious mental illness that requires effective treatment with minimal adverse effects. As preclinical and clinical research progresses, trace amine-associated receptor 1 (TAAR1) is becoming a potential new target for the treatment of schizophrenia.

**Methods:** We used molecular docking and molecular dynamics (MD) simulations to discover TAAR1 agonists. The agonistic or inhibitory effects of compounds on TAAR1, 5-HT1A, 5-HT2A, and dopamine D_2_-like receptors were determined. We used an MK801-induced schizophrenia-like behavior model to assess the potential antipsychotic effects of compounds. We also performed a catalepsy assay to detect the adverse effects. To evaluate the druggability of the compounds, we conducted evaluations of permeability and transporter substrates, liver microsomal stability *in vitro*, human ether-à-go-go-related gene (hERG), pharmacokinetics, and tissue distribution.

**Results:** We discovered two TAAR1 agonists: compounds 50A and 50B. The latter had high TAAR1 agonistic activity but no agonistic effect on dopamine D_2_-like receptors and demonstrated superior inhibition of MK801-induced schizophrenia-like behavior in mice. Interestingly, 50B had favorable druggability and the ability to penetrate the blood-brain barrier (BBB) without causing extrapyramidal symptoms (EPS), such as catalepsy in mice.

**Conclusion:** These results demonstrate the potential beneficial role of TAAR1 agonists in the treatment of schizophrenia. The discovery of a structurally novel TAAR1 agonist (50B) may provide valuable assistance in the development of new treatments for schizophrenia.

## Introduction

Schizophrenia is a complex and severe psychiatric disorder. The World Health Organization considers schizophrenia one of the top 10 disorders contributing to the global burden of disease, which affects >20 million people worldwide (Charlson et al., 2018).

The primary clinical symptoms of schizophrenia can be categorized into three groups: positive symptoms (characterized by hallucinations, delusions, and thought alterations); negative symptoms (avolition, aphasia, and impaired social interaction); and cognitive deficits (impaired executive functioning and memory) (van Os and Kapur, 2009). Due to the diversity and complexity of its symptoms, schizophrenia remains one of the most challenging diseases to manage.

Since the discovery of the antipsychotic drug (APD) chlorpromazine in 1952, APDs have elicited clinical benefits in patients with psychosis, presumably through antagonist or partial-agonist effects at postsynaptic dopamine D_2_-like receptors (Lieberman et al., 2005; Aringhieri et al., 2018; Girgis et al., 2019). However, the efficacy of dopamine D_2_-like receptors is limited in the treatment of negative symptoms, such as emotional retardation and aphasia, and cognitive impairment (Leucht et al., 2017; Millan et al., 2015). Atypical APDs with antagonist activity at dopamine D_2_ receptors and 5-hydroxytryptamine type 2A (5-HT_2A_) receptors have been shown to produce fewer side effects associated with dyskinesia. However, they have not shown significant improvements in efficacy and have increased the prevalence of adverse metabolic effects and body weight gain (Cooper et al., 2016; Taipale et al., 2018). Therefore, the discovery of APDs with a novel mechanism, high efficacy, and few side effects has become crucial.

In addition to the antagonism of dopamine D_2_-like and 5-HT_2A_ receptors, several other therapeutic targets have been studied extensively for their potential in treating schizophrenia, including receptors for α-amino-3-hydroxy-5-methyl-4-isoxazolepropionic acid, n-methyl-D-aspartic acid (NMDA), GlyT1, and muscarinic M_1_/M_4_ (Miyamoto et al., 2005; Girgis et al., 2019). However, they have shown limited or indifferent efficacy in clinical trials, except for SEP-856, agonism at trace amine-associated receptor 1 (TAAR1) (Karam et al., 2010; Girgis et al., 2019; Koblan et al., 2020), and KarXT (a drug containing xanomeline and trospium) (Brannan et al., 2021). KarXT elicits central antipsychotic effects without causing peripheral adverse effects, but strict control of the xanomeline:trospium ratio is required, which is not conducive to drug development.

TAAR1 is a member of the TAAR family located in the dorsal raphe nucleus and ventral tegmental area of the brain. Its activation partly inhibits dopamine D_2_-like receptors and activates 5-HT_1A_ receptors by regulating monoamine transmission and signaling (e.g., glycogen synthase kinase-3 beta signaling) (Miller, 2011; Revel et al., 2011; Harmeier et al., 2015). An agonist of the TAAR1 receptor, SEP-856, was discovered through an *in vivo* phenotypic drug discovery approach and has shown efficacy in phase-II clinical trials for treating schizophrenia. SEP-856 has been shown to reduce phencyclidine-induced hyperactivity and improve deficits in social interaction and pre-pulse inhibition in preclinical studies (Dedic et al., 2019; Saarinen et al., 2022). Another TAAR1 agonist, RG7906, is also in phase-II clinical trials for schizophrenia treatment. Therefore, TAAR1 may be an efficacious and safe target for schizophrenia treatment. Discovering more TAAR1-activating drugs may aid the development of efficacious and safe treatments for schizophrenia.

In the present study, two enantiomeric compounds were identified to have TAAR1 agonistic activity. Compound 50B activated TAAR1 better than SEP-856, as determined through molecular docking, molecular dynamics (MD) simulations, and functional assays on human TAAR1. Off-target assays revealed that 50B slightly activated 5-HT_1A_ and 5-HT_2A_ and had no excitatory or inhibitory effect on dopamine D_2_-like receptors, indicating that 50B may be less likely to elicit adverse effects. Pharmacokinetic studies and studies on blood–brain barrier (BBB) permeability demonstrated that 50B entered the brain in sufficient amounts. An *in vivo* pharmacodynamic experiment and catalepsy assay revealed that 50A and 50B reduced MK801-induced schizophrenia-like behavior in mice significantly without causing excessive extrapyramidal symptoms (EPS). Druggability analyses suggested that 50B had good BBB permeability and stability without cardiotoxicity. Overall, 50B, as a novel TAAR1 agonist, has good druggability, efficacy, and safety in schizophrenia treatment and may become a novel and powerful APD.

## Materials and methods

### Materials

Compounds 50A and 50B were supplied by Shanghai Wuxi Apptec Co., Ltd. (purity over 98.0%), and MS (ESI) m/z = 170.3 [M + H] +. SEP-856 (HY-136109A) and risperidone (HY-11018) were purchased from MedChemExpress (United States). Ham’s F-12K (Kaighn’s) Medium (11765070), Minimum Essential Medium (MEM, 11095080), Dulbecco’s modified Eagle medium/Nutrient Mixture F-12 (DMEM/F12, 11320033), and 0.25% Trypsin–EDTA solution (25200072) were purchased from Invitrogen (Camarillo, CA). MK801 was purchased from Sigma-Aldrich (M107) (St. Louis, United States). Fetal bovine serum (FBS) was purchased from Gibco (10099141C) (Carlsbad, CA). HitHunter^®^ cAMP Assay Kit (90-0075SM10) was obtained from Eurofins (Ontario, United States). FLIPR Calcium Assay Kit (R8194) was produced by Molecular Devices (California, United States).

### Ethical approval of the study protocol

All procedures related to animal experimentation were in accordance with Chinese laws and regulations governing the use and care of laboratory animals and the regulations set by our institution. The number of animals used, experimental design, and procedures for animal handling were approved by the Experimental Animal Ethics Committees of Yantai University.

### Animals

Male Sprague–Dawley rats (6–8 weeks; 190–210 g) obtained for studies on pharmacokinetics and tissue distribution and male C57BI/6J mice (6–8 weeks; 18–22 g) used for the pharmacokinetics study were purchased from Pengyue Laboratory Animal Technology (Jinan, China). All animals were kept in a standard environment (20°C–23°C; 12 h light–dark cycle; relative humidity = 60%–65%).

### Molecular docking

Binding analyses of compounds attached to TAAR1 were conducted using the LibDock module in Discovery Studio 2018 (https://discover.3ds.com/discovery-studio/). As of 16 August 2021, the crystal structure of TAAR1 was unavailable in the RCSB Protein Data Bank (www.pdbus.org/). Hence, homology modeling was used to generate its three-dimensional structure using AlphaFold (https://alphafold.ebi.ac.uk/entry/Q96RJ0/). The target was optimized for dehydration, hydrogenation, protonation, and energy minimization. The active site in TAAR1 was defined based on the verified contact residues Asp103 (Nair et al., 2022). All compounds were prepared for docking simulation to consider appropriate protonation states, charges, and energy minimization.

### MD simulations

The dynamic behavior of compounds and TAAR1 was studied by MD simulations on Gromacs 5.1.4 (Abraham et al., 2015). By taking the TAAR1–50B simulation as an example, the target was modeled with the amber99sb-ildn force field (Lindorff-Larsen et al., 2010), and the ligand with a charge state of +1 was parameterized with a GAFF force field in AMBER14 (Case et al., 2014). The solvation of the system was conducted with TIP3P water molecules in a cubic box with a margin of 1.2 nm, and counterions were added to neutralize the system. During MD simulations, the system converged to a minimum energy level using the steepest-descent method of 50,000 steps and force <10.0 kJ/mol. Subsequently, one equilibration simulation under the constant volume (NVT) ensemble was conducted with 100 ps, followed by 100 ps with constant pressure (NPT) equilibration. Subsequently, a routine MD simulation for 100 ns was undertaken without restraints. The root mean square deviation (RMSD) and root mean square fluctuation (RMSF) of the trajectory were calculated using “Gromacs” tools.

### Cyclic adenosine monophosphate (cAMP) assay to determine the functional activity/inhibition at human TAAR1 receptors and dopamine D_2L_ receptors

With respect to TAAR1, cAMP Hunter™ Chinese hamster ovary (CHO)-K1 AGTRL1 Gi cells expressing human TAAR1 receptors on cell membranes were cultured with Ham’s F-12K (Kaighn’s) medium supplemented with 10% FBS, penicillin (100 U/ml), and streptomycin (100 μg/ml) at 37°C in an atmosphere of 95% air and 5% CO_2_. Cells were seeded in a total volume of 20 µL into white-walled, 384-well microplates, followed by incubation at 37°C for the appropriate time before testing. Different concentrations of compounds were added to cells, and incubation was undertaken at 37°C for 10 min, with *p*-tyramine used as the MAX control. cAMP modulation was determined using the DiscoverX HitHunter cAMP XS + assay. Percentage activity was calculated using the following equation:

$$Activity\;\left(\%\right)=\left(mean\;{RLU}_{test\;sample}\;–\;mean\;{RLU}_{vehicle\;control}\right)/\left(mean\;{RLU}_{\mathrm{MAX}\;control}\;–\;mean\;{RLU}_{vehicle\;control}\right)\times 100\%.$$

With regard to D_2L_ and D_2S_, recombinant Flp-In™-CHO cells expressing human dopamine D_2L_ receptors or D_2S_ receptors were cultured with Ham’s F-12K (Kaighn’s) medium supplemented with 10% FBS, penicillin (100 U/ml), streptomycin (100 μg/ml), and hygromycin B (600 μg/ml) at 37°C in an atmosphere of 5% CO_2_. The methods of cell inoculation and compound treatment were the same as those for TAAR1, and haloperidol was used as the MAX control. Percentage inhibition was calculated using the following equation:

$$Inhibition\;\left(\%\right)=\left(mean\;{RLU}_{test\;sample}-mean\;{RLU}_{vehicle\;control}\right)/\left(mean\;{RLU}_{\mathrm{MAX}\;control}-mean\;{RLU}_{vehicle\;control}\right)\times 100\%.$$

### Calcium assay to determine functional activity at human 5-HT_1A_ and 5-HT_2A_ receptors

With respect to 5-HT_1A_, recombinant Gα15-CHO-Clone cells expressing human 5-HT_1A_ were used in this test. Cells were cultured with MEM supplemented with 10% FBS, penicillin (100 U/ml), streptomycin (100 μg/ml), and hygromycin B (600 μg/ml) and were seeded in 384-well microplates. Cells were stimulated with different concentrations of compounds for 2 h at 37°C, and the agonistic effect was determined using the FLIPR Calcium Assay Kit. Serotonin was used as the MAX control. Percentage activity was calculated using the following equation:

$$Activity\;\left(\%\right)=\left(mean\;{RLU}_{test\;sample}-mean\;{RLU}_{vehicle\;control}\right)/\left(mean\;{RLU}_{\mathrm{MAX}\;control}-mean\;{RLU}_{vehicle\;control}\right)\times 100\%.$$

With regard to 5-HT_2A_, the agonistic or inhibitory activity of 5-HT_2A_ of compounds was determined using the Flp-In™-CHO cell line expressing human 5-HT_2A_. Cells were cultured with Ham’s F-12K (Kaighn’s) medium supplemented with 10% FBS, penicillin (100 U/ml), streptomycin (100 μg/ml), and hygromycin B (600 μg/ml) at 37°C in an atmosphere of 95% air and 5% CO_2_. Cells were stimulated with different concentrations of compounds for 2 h at 37°C after seeding in 384-well microplates. For the determination of agonistic activity, serotonin was used as the MAX control. In the determination of inhibitory activity, ketanserin was used as the MAX control.

### Evaluation of permeability and transporter substrates

Caco-2 cells were cultured with MEM supplemented with 20% FBS, penicillin (100 U/ml), and streptomycin (100 μg/ml) at 37°C in an atmosphere of 95% air and 5% CO_2_. Cells were seeded into 96-well cell plates and cultured continuously for 24 days for subsequent experiments. Compounds were administered with or without verapamil (2 µM). After incubation for 120 min, samples at the apical and basal ends were collected. The urea content in each sample was measured by liquid chromatography–tandem mass spectrometry (LC-MS/MS). The apparent permeability coefficient and the efflux ratio were calculated.

### Evaluation of liver microsomal stability *in vitro*

Compounds were incubated with samples of liver microsomes from mice, rats, and humans and NADPH for 5, 10, 20, 30, or 60 min at 37°C. A cold acetonitrile solution containing tolbutamide was used to terminate the reaction. Testosterone and dextromethorphan were used as positive controls. LC-MS/MS was employed to determine the retention of compounds. The retention time, chromatogram acquisition, and chromatogram integration of compounds and positive control substances were analyzed using Analyst (AB Sciex, Framingham, MA, United States).

### Human ether-à-go-go-related gene (hERG) assay

CHO cells stably expressing hERG cultured with DMEM/F12 supplemented with 10% FBS, penicillin (100 U/ml), and streptomycin (100 μg/ml) were cultured in cell culture dishes (diameter = 35 mm) in an incubator at 37°C in an atmosphere of 5% CO_2_. They were passaged at a ratio of 1:5 every 48 h. The medium comprised 90% F12, 10% FBS, G418 (100 μg/ml), and hygromycin B (100 μg/ml). On the day of the assay, the cell culture medium was aspirated and washed with an extracellular solution. Then, 0.25% Trypsin–EDTA solution was added, and digestion was allowed for 3–5 min at room temperature. Digestive juice was aspirated, and cells were transferred to the experimental dish for electrophysiological recording after resuspension with the extracellular solution for future use.

### Pharmacokinetics study

Compound 50B was dissolved in physiologic (0.9%) saline. Male and female rats were fasted overnight before dosing to avoid the possible effects of food. Compound 50B was administered via intragastric (5 mg/kg) and intravenous (2 mg/kg) routes to female and male rats. Approximately 200 μL of whole blood was collected into heparin-coated tubes at 0.083, 0.25, 0.5, 1, 2, 3, 4, 6, 8, 12, and 24 h after dosing. Plasma was collected immediately by centrifugation (8,000 g, 5 min, room temperature). Blood was placed in a heparinized Eppendorf™ tube. Plasma was centrifuged, and the concentration of compounds in the sample was determined by LC-MS/MS after pre-treatment. Drug-concentration data were processed in a non-atrioventricular model using WinNonlin™ 6.3 (Pharsight, Mountain View, CA, United States).

### Tissue distribution

Rats (three males and three females) were sacrificed at 0.25, 1, or 6 h after administration of 50B (5 mg/kg, i.g.). Blood and brain tissues were harvested. The concentration of compounds in all tissue and blood samples was determined by LC-MS/MS. Drug concentration data were processed in a non-atrioventricular model using WinNonlin™ 6.3.

### MK801-induced schizophrenia-like behavior

Compounds 50A, 50B, and SEP-856 were dissolved in physiologic saline. Risperidone and aripiprazole were dissolved in a 0.5% sodium carboxymethyl cellulose (Na-CMC) solution.

Ninety-nine male C57BI/6J mice were divided randomly into 11 groups. Mice were placed in a room 1 h before testing. They were administered (p.o.) vehicle (0.1 ml/10 g), 50A (0.3, 1, 3, and 10 mg/kg; 0.1 ml/10 g), or 50B (0.3, 1, 3, and 10 mg/kg; 0.1 ml/10 g). Mice were placed in open-field chambers (27.3 × 27.3 × 20.3 cm; Med Associates, Saint Albans, VT, United States) for 30 min to measure the baseline activity of compounds. Then, mice were administered (i.p.) MK801 (0.8 mg/kg dissolved in physiologic saline; 0.1 ml/10 g). After 30 min, the distance that mice traveled was measured.

Forty-six male C57BI/6J mice were divided randomly into four groups. The CN group contained four mice. Ten mice were in the vehicle group (0.1 ml/10 g, p.o.). Eight mice were in the SEP-856 group (10 mg/kg, p.o.; 0.1 ml/10 g), 50B group (10 mg/kg, p.o.; 0.1 ml/10 g), risperidone group (0.1 mg/kg, p.o.; 0.1 ml/g) group, and aripiprazole group (5 mg/kg, p.o.; 0.1 ml/10 g), respectively. The administration method described previously was employed.

### Catalepsy assay

Twenty-four male C57BI/6J mice were divided randomly into four groups. Mice were placed in a room 1 h before testing. They were administered vehicle, risperidone (1 or 5 mg/kg, p.o.; 0.1 ml/10 g), 50A (100 mg/kg, p.o.; 0.1 ml/10 g), or 50B (100 mg/kg, p.o.; 0.1 ml/10 g). The front paws of mice were placed on a horizontal metal bar raised 2 inches above a Plexiglas™ platform 30 min and 90 min after administration. The time the front paws of mice were placed on the platform was recorded at ≤30 s per trial. Each test was repeated three times.

### Statistical analyses

Statistical analysis was undertaken using Prism 8 (GraphPad, La Jolla, CA, United States). The results are the mean ± SD. For data with a single dose or time point, the differences between groups were analyzed by non-parametric ANOVA. A *p*-value of 0.05 or less was considered statistically significant.

## Results

### Structural models of TAAR1 and binding of 50A and 50B

**FIGURE 1:**
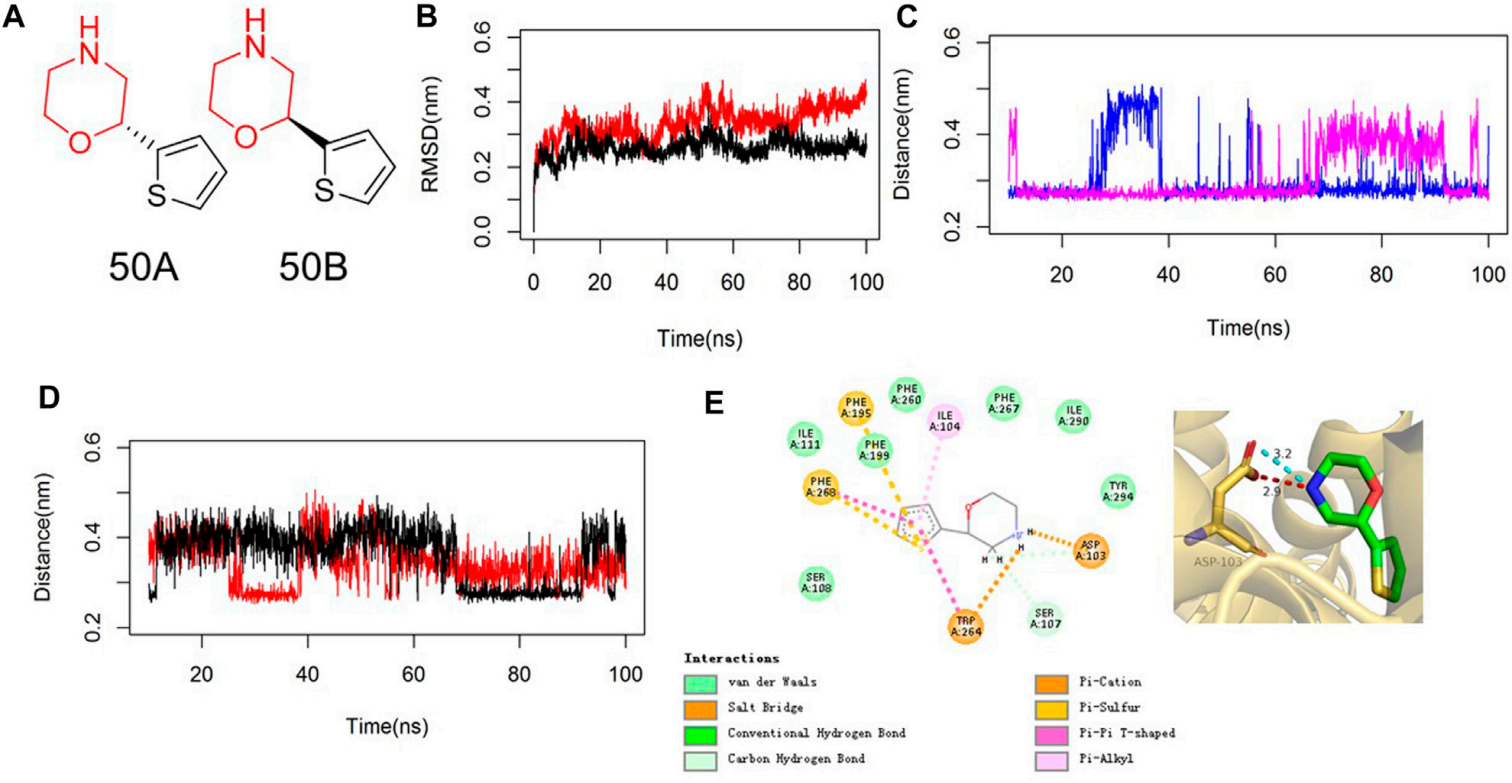
MD analyses of 50B bound to TAAR1. **(A)** Chemical structure of 50A and 50B. **(B)** RMSD values of 50B (red) or 50A (black) bound to TAAR1. RMSD values are calculated from the backbone after the least squares fit to the backbone. **(C)** Salt-bridge distance of the N atom of morpholine in 50B (blue) or 50A (magenta) with Asp103 in TAAR1. **(D)** H-bond distance of –NH of 50B (red) or 50A (black) with the side-chain carbonyl O of Asp103 in TAAR1. **(E)** 2D (left) and 3D (right) plots of the representative conformation of 50B binding with TAAR1. The key residues in TAAR1 and 50B are shown in golden and green sticks, respectively. The hydrogen bond and electrostatic interaction are shown in red and cyan dashed lines, respectively.

Asp103 in TAAR1 is conserved in all G protein-coupled receptors that bind biogenic amines so that diverse physiologic functions can be undertaken. Nair et al. (2022) reported that the side-chain carboxylate O^−^ of Asp103 in TAAR1 can form a salt-bridge bond with the positively charged N atom of SEP-856, which is beneficial for the antipsychotic activity. We undertook similarity searching to find compounds with similarity >0.7 using MACCS keys and the Tanimoto Index. Then, 50B and 50A with a high similarity score were modeled using molecular docking and MD simulations. The RMSD value by MD simulations showed that compound 50B reached a relatively stable position after achieving equilibrium in the MD trajectory (Figure 1B), and >92% conformations demonstrated that the N atom of morpholine in 50B formed a salt-bridge bond with Asp103 with a bond length ranging from 0.25 nm to 0.45 nm (Figure 1C). Moreover, the –NH group of 50B formed a stable H-bond (distance ∼0.31 nm) with the side-chain carbonyl O of Asp103 (Figure 1D). The thiophene moiety of 50B participated in π–π stacking interactions with Phe195, Phe268, and Trp264 (Figure 1E). In contrast, the MD simulation of 50A bound to TAAR1 showed large fluctuations in the distance of the –NH group in 50A and side-chain O atom of Asp103. Beginning from ∼11 ns to the MD simulation, the –NH group of 50A pointed toward the carboxylate O^−^ of Asp103. From about 65 ns, the–NH group of 50A moved closer to the carbonyl O and away from carboxylate O^−^ of Asp103 (distance ∼0.40 nm), which weakened the potency of the agonist to the target markedly (Figures 1C,D). Accordingly, we proposed that the salt-bridge bond contributed by Asp103 was the predominant interaction with 50B.

### 50A and 50B had an agonistic effect on TAAR1

**FIGURE 2:**
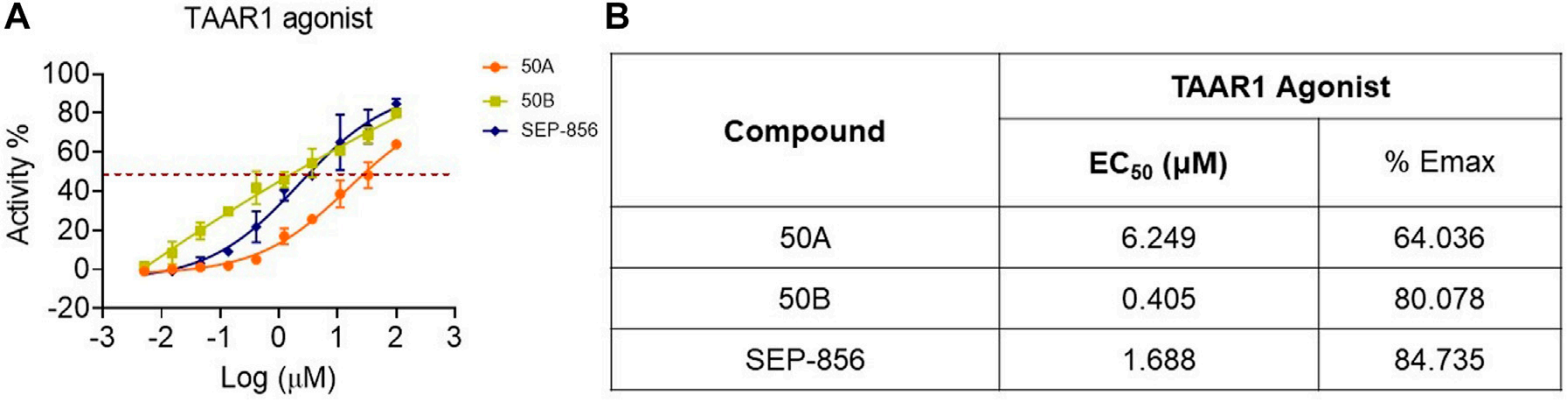
Functional effect of SEP-856, 50A, and 50B at TAAR1. **(A, B)** The TAAR1 functional effect assays were determined using CHO-K1 AGTRL1 Gi cells expressing human TAAR1 on the cell membrane, and cAMP modulation was determined. Every assay was repeated three times.

To discover more TAAR1 agonists, we conducted functional tests using cAMP Hunter™ CHO-K1 AGTRL1 Gi cells with membrane overexpression of human TAAR1. 50A and 50B activated TAAR1, and the half-maximal effective concentration (EC_50_) of 50B on TAAR1 reached 0.405 μM, significantly lower than that of SEP-856 (Figures 2A,B). These findings indicated that 50A and 50B were novel agonists at human TAAR1.

### 50B slightly activated 5-HT_1A_ and 5-HT_2A_ receptors and had no excitatory or inhibitory effects on dopamine D_2_-like receptors

**FIGURE 3:**
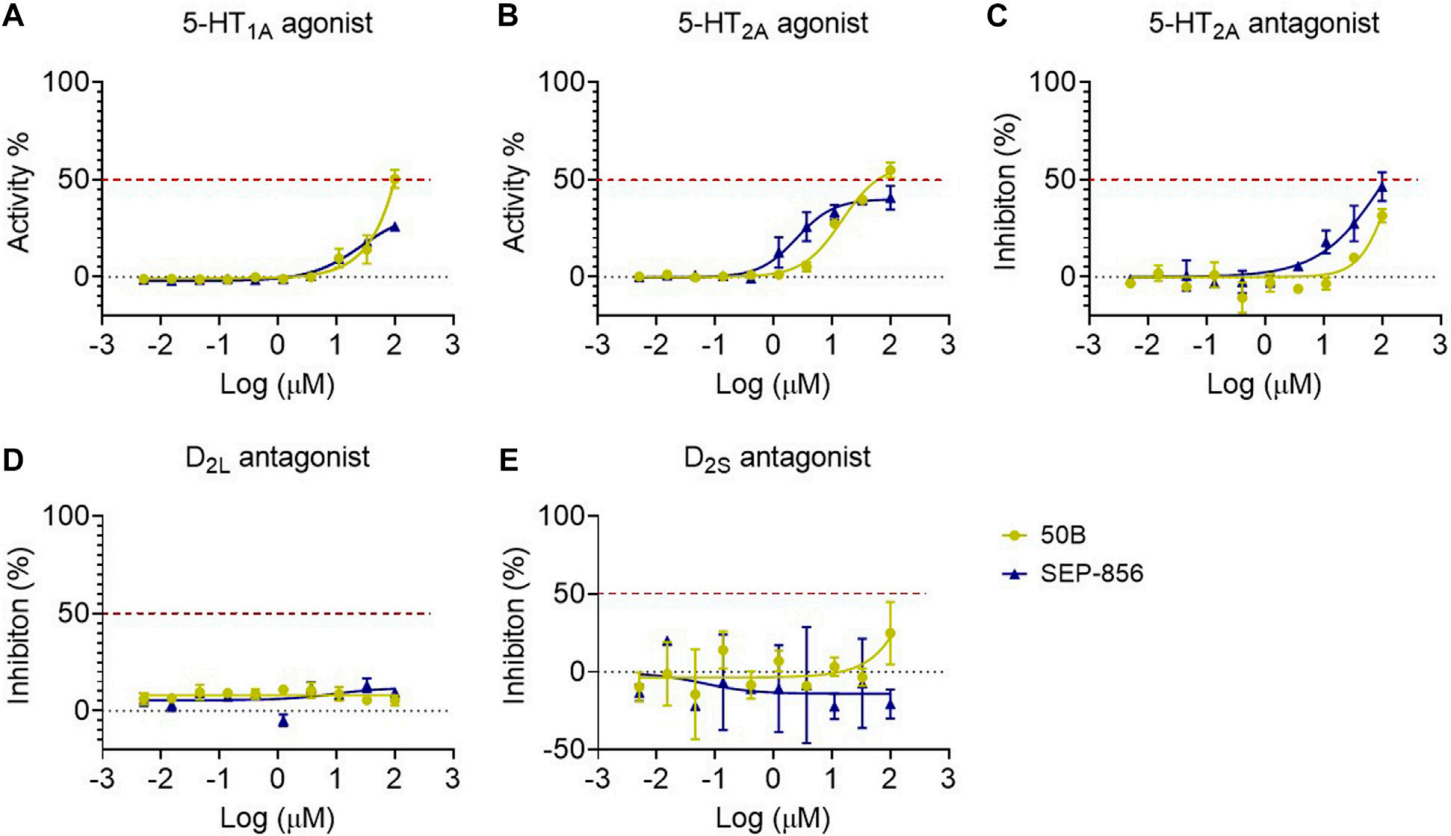
Agonist–antagonist activity of SEP-856 and 50B at 5-HT_1A_, 5-HT_2A_, dopamine D_2L_, and D_2S_ receptors. **(A)** The 5-HT_1A_ receptor functional effect assay was determined using recombinant Gα15-CHO-Clone cells expressing human 5-HT_1A_ receptor, and the agonist effect was determined using the FLIPR Calcium Assay Kit. **(B, C)** The agonist or antagonist activity of 5-HT_2A_ receptors of the compounds was performed using Flp-In™-CHO Cell Lines expressing human 5-HT_2A_ receptors. The functional effect was also assessed via the FLIPR Calcium Assay Kit. **(D, E)** The dopamine D_2L_ and D_2S_ receptors’ functional effect assays were determined using recombinant Flp-In™-CHO cells expressing human dopamine D_2L_ receptors. Accumulation of cAMP was determined using the DiscoverX HitHunter cAMP XS + assay. Every assay was repeated three times.

Functional testing revealed that 50B (100 μM) was a weak partial agonism on 5-HT_1A_ (EC_50_ = 99.77 μM and E_max_ = 50.33%) and 5-HT_2A_ (EC_50_ = 61.23 μM and E_max_ = 54.88%) receptors (Figures 3A,B) but showed no antagonist activity on 5-HT_2A_ (IC_50_ > 100 μM and E_max_ = 31.49%), dopamine D_2L_ (IC_50_ > 100 μM and E_max_ = 5.49%), and dopamine D_2S_ (IC_50_ > 100 μM and E_max_ = 24.93%) receptors (Figures 3C–E).

### 50B had favorable pharmacokinetic characteristics and effectively crossed the BBB

**FIGURE 4:**
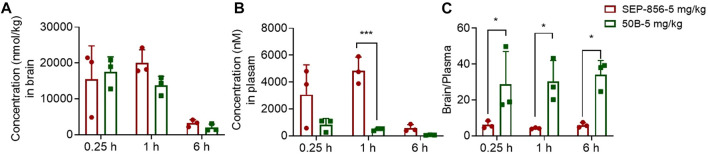
Concentrations of 50B and SEP-856 in brain and plasma, as determined by LC-MS/MS. **(A)** Concentration–time profiles of 50B and SEP-856 in the brain. **(B)** Concentration–time profiles of 50B and SEP-856 in plasma. **(C)** Concentration ratio of 50B and SEP-856 in the brain to plasma. Results are expressed as mean ± SD (*n* = 3). ^*^ *p* < 0.05 and ^***^ *p* < 0.001.

To analyze the druggability of 50B further, we characterized its pharmacokinetic characteristics and ability to cross the BBB. After single intragastric administration of 50B (5 mg/kg), the time-to-peak concentration was ∼0.833 h, the mean elimination half-life was ∼1.28 h, the concentration–time curve from 0 to time *t* was ∼18,929 h.nM, and oral bioavailability was ∼84.3% (Table 1). Good permeability through the BBB is conducive to the development of APDs, so the intracerebral distribution of 50B was measured. After single intragastric administration of 50B (5 mg/kg) or the positive control (SEP-856, 5 mg/kg), 50B or SEP-856 was detected in rat brains from 0.25 to 6 h (Figure 4A). Interestingly, at the same time point after intragastric administration, although the plasma concentration of 50B was lower than that of SEP-856 (time × drug interaction, F_(1, 12)_ = 24.39, *p* < 0.001) (Figure 4B), the concentration ratio of 50B in the brain to plasma increased significantly compared with that of SEP-856, which indicated that 50B had greater permeability through the BBB (time × drug interaction, F_(1,12)_ = 32.35, *p* < 0.001) (Figure 4C).

**TABLE 1:** Pharmacokinetics of 50B in male rats.

Compound	T_max_ (h)	C_max_ (nM)	AUC_0-t_ (h* nM)	T_1/2_ (h)	V_z_/F (L/kg)	C_L_/F (L/h/kg)	MRT_last_ (h)	F (%)
50B (2 mg/kg, i.v.)	—	6,801 ± 1,464	8,980 ± 1,340	1.31 ± 0.313	2.48 ± 0.485	1.33 ± 0.211	1.46 ± 0.231	—
50B (5 mg/kg, i.g.)	0.833 ± 0.289	4,380 ± 1,688	18,929 ± 1774	1.28 ± 0.140	2.86 ± 0.104	1.56 ± 0.146	3.03 ± 0.625	84.3 ± 7.90

### 50A and 50B significantly alleviated MK801-induced schizophrenia-like behavior

**FIGURE 5:**
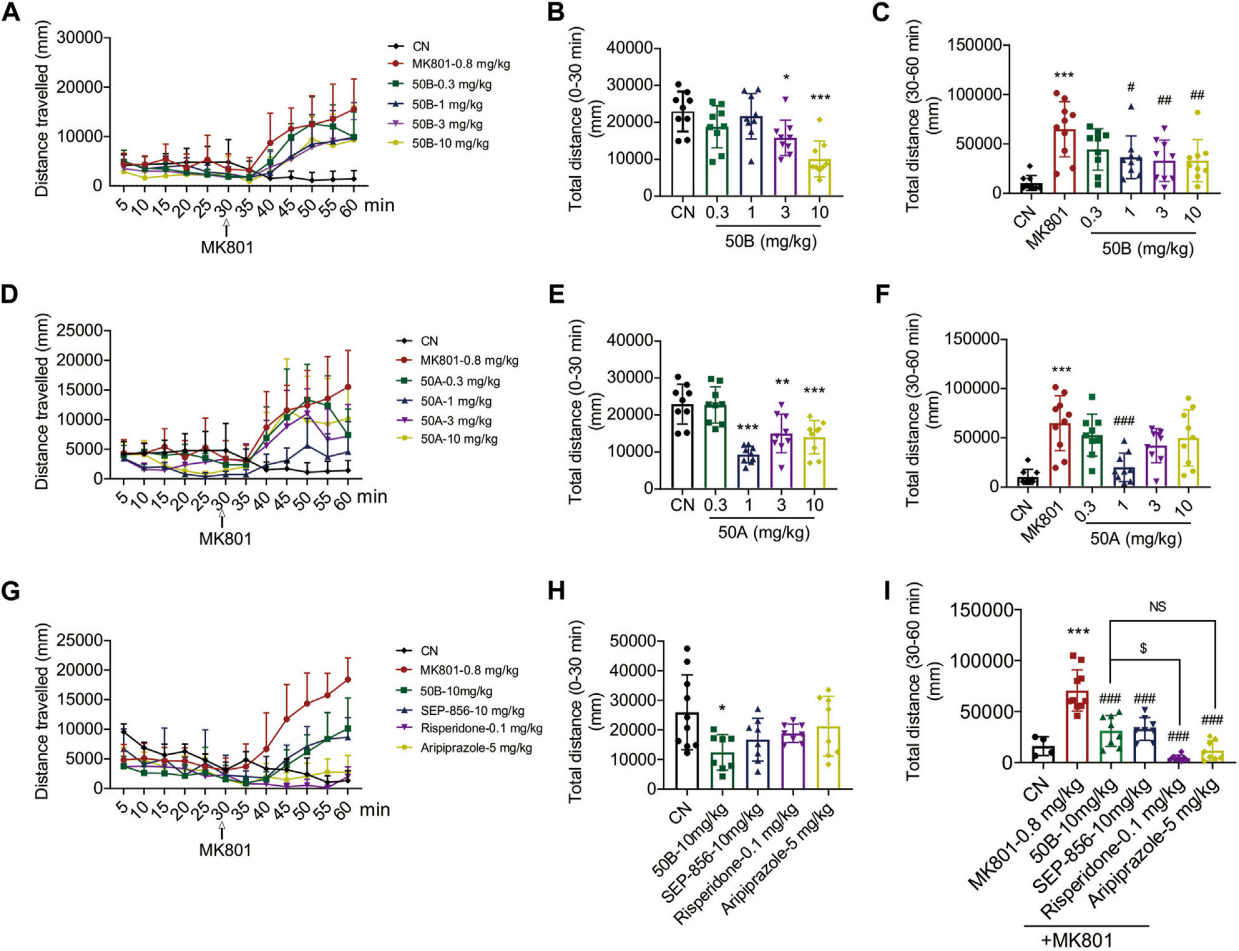
50B and 50A improved MK801-induced schizophrenia-like behavior, and 50B had similar effects compared with positive control (aripiprazole). **(A–F)** Oral 50B or 50A administration dose-dependently reduced MK801-induced schizophrenia-like behavior in C57BL/6J mice. Results are expressed as mean ± SD (n = 9). **(G–I)** Risperidone or aripiprazole also reduced MK801-induced schizophrenia-like behavior, 50B had a lower impact on reducing the distance mice traveled compared with risperidone, but had similar effects compared with aripiprazole. Results are expressed as mean ± SD (n = 4 or 8). ^*^ *p* < 0.05, ^**^ *p* < 0.01, and ^***^ *p* < 0.001 *versus* the control group (CN); ^#^ *p* < 0.05, ^##^ *p* < 0.01, and ^###^ *p* < 0.001 *versus* the MK801 group; $*p* < 0.05.

An antagonist of the NMDA receptor, MK801, is commonly used to induce psychosis-like symptoms in mice and is an important tool for screening APDs (Kruk-Slomka et al., 2017; Okuyama et al., 2017; Fan et al., 2018). To assess the potential antipsychotic effects of 50A and 50B, we administered a single oral dose of these compounds (0.3, 1, 3, and 10 mg/kg) to C57BI/6J mice in the presence or absence of MK801. Treatment with 50A or 50B significantly reduced the distance moved by mice in the presence or absence of MK801. Therapy with 50A inhibited MK801-induced schizophrenia-like behavior only at 1 mg/kg (F_(4, 40)_ = 14.85, *p* < 0.001 (Figure 5E); F_(5, 49)_ = 9.037, *p* < 0.001 (Figure 5F)), whereas 50B treatment inhibited MK801-induced schizophrenia-like behavior markedly at 1, 3, and 10 mg/kg (F_(4, 40)_ = 8.152, *p* < 0.001 (Figure 5B); F_(5, 49)_ = 6.692, *p* < 0.001 (Figure 5C)).

The potential of 50B and SEP-856 in ameliorating schizophrenia-like behavior induced by MK801 was also assessed. Administration of 50B or SEP-856 at 10 mg/kg, respectively, significantly reduced MK801-induced increase in distance traveled by mice, and there was no significant difference between the two groups (F_(4, 37)_ = 2.943, *p* = 0.033 (Figure 5H); F_(5, 40)_ = 28.22, *p* < 0.001 (Figure 5I)). Treatment with the positive control, risperidone, or aripiprazole also significantly reduced MK801-induced schizophrenia-like behavior (Figures 5H,I). Compared with risperidone, 50B had a lower impact on reducing the distance mice traveled but had similar effects compared with aripiprazole (Figure 5I).

### 50A and 50B had no effect on the time mice spent holding bars

**FIGURE 6:**
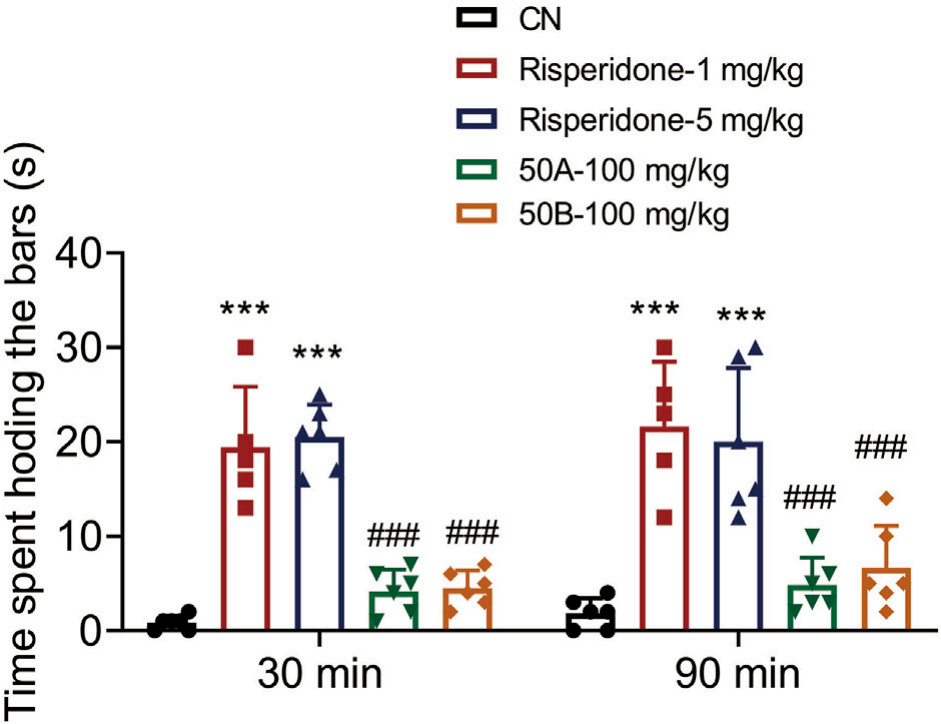
50A and 50B did not induce catalepsy in male mouse bar test. Results are expressed as mean ± SD (*n* = 5–6). ^***^ *p* < 0.001 *versus* the control group (CN); ^###^ *p* < 0.001 *versus* 1 mg/kg of the risperidone group.

One of the most serious adverse effects of APDs is EPS, which significantly affects the daily activities of patients. EPS can be assessed in mice by measuring the induction of catalepsy with bar tests. Risperidone (1, 5 mg/kg) treatment increased the time mice spent holding bars significantly, whereas 50A and 50B at 100 mg/kg, respectively, did not increase this time. Hence, 50A and 50B did not cause EPS at doses ≥10 times the effective dose (time × drug interaction, F_(4, 48)_ = 50.14, *p* < 0.001) (Figure 6). 50A and 50B, as novel TAAR1 agonists, may be efficacious and safe for schizophrenia treatment.

### 50B had the valuable characteristic of druggability

The results stated previously suggested that as a novel agonist of TAAR1, 50B flowed to brain and had a role in the anti-positive symptoms of schizophrenia. Therefore, we further analyzed its druggability. Membrane permeability, microsomal metabolism, and cardiotoxicity of 50B were measured. 50B showed strong membrane permeability and good microsomal metabolic stability (Tables 2, 3), similar to those of the positive control SEP-856. The hERG assay also showed that 50B was not cardiotoxic up to a maximum concentration of 30 μM. Overall, these results supported further development and investigation of 50B as a potential treatment for schizophrenia.

**TABLE 2:** Membrane permeability of 50B and SEP-856.

Compound	Mean P_app_ (10^−6 ^cm/s)		Efflux ratio
	A to B	B to A	
50B	35.2	27.5	0.781
SEP-856	40.4	34.3	0.850

**TABLE 3:** Microsomal metabolic stability in liver microsomes from different species.

Compound	T_1/2_ (min)			CL_int_ (liver) (ml/min/kg)			Remaining (%, T = 60 min)		
	Human	Mouse	Monkey	Human	Mouse	Monkey	Human	Mouse	Monkey
50B	161	67.9	>186	7.74	80.8	<13.5	77.2	53.5	85.1
SEP-856	>186	>186	>186	<6.75	<29.7	<13.5	91.8	88.8	85.7

## Discussion

The etiology of schizophrenia is unknown, but many of its symptoms can be alleviated by treatment with various APDs. The most recent are third-generation partial dopamine D_2_-like receptors agonist APDs. Third-generation APDs are effective in relieving positive symptoms without causing hyperprolactinemia and have a lower EPS burden compared to first-generation APDs and lower weight gain and metabolic burden compared to second-generation APDs (Gomez-Revuelta et al., 2021; Chen et al., 2022). Despite improvements in drug safety, third-generation APDs still work on dopamine D_2_-like and the 5-HT receptors. Adverse effects, such as EPS, are reduced but are still unavoidable. In addition, approximately 30% of the patients have treatment-resistant schizophrenia. Thus, the urgency for new therapies is evident.

New APDs focusing on new targets have been developed but have not been successful (although GlyT1 inhibition and agonism at mGlu2/3 receptors have been successful in non-clinical and clinical proof-of-concept studies) (Marsman et al., 2013; Kinon et al., 2015; Girgis et al., 2019; Pahwa et al., 2021). A clinical trial showed that the mean change in the total score of the positive and negative syndrome scale of the patients with schizophrenia in the non-D_2_-like receptor antagonist (SEP-856) group was −7.2 points after 4 weeks of treatment, which was significantly lower than the −9.7 points in the placebo group. With regard to safety, patients in the SEP-856 group experienced major adverse events (e.g., somnolence and gastrointestinal symptoms). However, the prevalence of EPS and changes in blood levels of lipids, glycosylated hemoglobin, and prolactin were similar to those in the placebo group (Koblan et al., 2020). These results suggest SEP-856 to be a valuable option in schizophrenia treatment and to avoid the side effects of direct antagonism of dopamine D_2_-like receptors.

Mechanistic studies have shown that TAAR1 activation is involved in the antipsychotic effect of SEP-856. TAAR1 is activated by trace amounts (TAs) of amines (Bunzow et al., 2001). TAAR1 is widely expressed in the mammalian brain (especially in limbic and monoaminergic regions) and is associated with mood, attention, memory, fear, and addiction (Rutigliano et al., 2017; Xu and Li, 2020). In the periphery, TAAR1 is expressed in small amounts in the thyroid, gastric, and pancreatic glands. However, its role in the brain is not known.

Several studies have investigated the possible link between schizophrenia and TA disorders in urine, cerebrospinal fluid, and plasma. TAs of amines are significantly reduced in the brains of patients with various neurologic disorders, which may suggest that the downregulation of TAAR1 function is related to the pathogenesis of psychiatric disorders (Gainetdinov et al., 2018; Dedic et al., 2019; Dodd et al., 2021). However, other studies have shown the low concentrations and rapid turnover (<30 s) of TAs of palmitoylethanolamide (PEA), tryptamine, and octopamine to be associated with a lack of vesicular storage (Berry, 2004).

Studies have shown that SEP-856 suppressed aberrant neuronal discharge in the ventral tegmental region of the midbrain and reduced ketamine-induced dopamine production in the striatum of mice (Begni et al., 2021; Dedic et al., 2019). Recent studies have reported SEP-856 to have a significant inhibitory effect on baseline locomotion in wild-type mice but not in TAAR1-knockout mice. Furthermore, the ability of SEP-856 to increase sensorimotor gating depends on TAAR1 (Saarinen et al., 2022). These results demonstrated the role of TAAR1 agonism in schizophrenia treatment. Therefore, the search for more novel activators of TAAR1 and related pharmacologic and pharmacogenetic studies are important for the development of therapeutic strategies for clinical schizophrenia.

We employed molecular docking to analyze the binding of small-molecule compounds to TAAR1. More than 1,000 compounds, with molecular weight <200 and similarity >0.5 with SEP-856, were selected for molecular docking. Compounds 50A and 50B had a high molecular docking score. They were selected to analyze the binding mode with TAAR1 using MD: 50A and 50B could bind to TAAR1. MD simulations showed that the binding of 50B to TAAR1 was strong and not altered significantly by molecular changes. The N atom of morpholine in 50B formed a salt-bridge bond with Asp103 with a bond length of 0.25–0.45 nm. Moreover, the –NH group of 50B formed a stable H-bond (distance ∼0.31 nm) with the side-chain carbonyl O of Asp103. The thiophene moiety of 50B participated in π–π stacking interactions with Phe195, Phe268, and Trp264.

Subsequently, the functional effects of 50A and 50B on TAAR1 activation were investigated. 50B significantly promoted the cAMP accumulation of CHO-K1 AGTRL1 Gi cells expressing human TAAR1 receptors on cell membranes (EC_50_ = 0.405 μM and E_max_ = 80.078%). 50B had weak 5-HT_1A_ and 5-HT_2A_ receptor agonistic activity (EC_50_ = 99.77 and 61.23 μM and E_max_ = 50.33% and 54.88%, respectively) and weak 5-HT_2A_ antagonistic activity (EC_50_ > 100 μM and E_max_ = 31.49%, respectively) and had no inhibitory effect on either isoform of dopamine D_2_-like receptors (D_2S_ or D_2L_). The off-target results stated previously showed that 50B was a relatively pure TAAR1 activator and had weak agonistic or antagonistic effects on the 5-HT and dopamine systems.

The highly selective activation of TAAR1 by 50B suggested its potential as an antipsychotic agent. To investigate this possibility, we analyzed the pharmacokinetic characteristics, BBB permeability, inhibition of MK801-induced schizophrenia-like behavior, and adverse effects of 50B. In MK801-induced schizophrenia-like behavior, 50A or 50B were given 30 min before MK801 administration. Treatment with 50A or 50B could significantly lessen the increase in distance traveled by mice caused by MK801. 50A (1 mg/kg) reduced the distance traveled by mice after MK801 stimulation, and 50B (1, 3, and 10 mg/kg) significantly reduced the distance traveled by mice after MK801 stimulation. Comparison of efficacy showed SEP-856 to have effects similar to those of 50B for suppressing MK801-induced schizophrenia-related symptoms. These data suggested that the superior role of 50B in resistance to the positive symptoms of schizophrenia is caused by its stronger activation of TAAR1. The agonistic activity of 50B on TAAR1 was superior to that of SEP-856. However, the plasma concentration of 50B was significantly lower than that of SEP-856 at the same time point after oral administration at 5 mg/kg. Thus, 50B and SEP-856 had similar effects in reducing the distance traveled by mice, which suggests that TAAR1 activation is involved in how 50B counteracts the positive symptoms of schizophrenia.

To further analyze the role of TAAR1 agonists in improving schizophrenia-like behavior, the positive compounds, second-generation APD (risperidone), and third-generation APD (aripiprazole) were used. Our results show that 50B had a lower impact on reducing the distance mice traveled compared with second-generation APD but had similar effects compared with third-generation APD, which suggested that 50B plays a comparable role in suppressing positive symptoms of schizophrenia to third-generation APD. In terms of inducing EPS, 50B therapy did not increase the time mice spent holding bars in the catalepsy assay. Catalepsy symptoms were found at a risperidone dose of 1 and 5 mg/kg, whereas 50A and 50B did not cause catalepsy symptoms 10 times the effective dose (100 mg/kg). These data suggest that TAAR1 agonists are less likely to cause EPS compared with risperidone. Further analysis of the druggability of 50B revealed strong permeability through the BBB, good microsomal metabolic stability, and no cardiotoxicity in the hERG assay.

## Conclusion

Our study demonstrated the role of TAAR1 activation in schizophrenia treatment. The TAAR1 agonist 50B significantly improved the positive symptoms of schizophrenia induced by MK801 in mice. 50B had favorable safety and efficacy. 50B may become a candidate compound for schizophrenia treatment.

## Data Availability

The raw data supporting the conclusion of this article will be made available by the authors, without undue reservation.
